# A prospective study of weight development and behavior problems in toddlers: the Norwegian Mother and Child Cohort Study

**DOI:** 10.1186/1471-2458-10-626

**Published:** 2010-10-20

**Authors:** Susan Garthus-Niegel, Knut A Hagtvet, Margarete E Vollrath

**Affiliations:** 1 Department of Psychosomatics and Health Behavior, Norwegian Institute of Public Health, Postbox 4404, Nydalen, 0403 Oslo, Norway; 2 HØKH, Research Centre, Akershus University Hospital, Box 95, 1478 Nordbyhagen, Norway; 3 Institute of Psychology, University of Oslo, Postboks 1094, Blindern, 0317 Oslo, Norway

## Abstract

**Background:**

Previous research has suggested that overweight children have a higher risk of behavior problems, but the causal direction of this relationship remains unclear. In a large prospective population study, we investigated whether child behavior problems and body mass index are associated in toddlers and whether overweight is a risk for behavior problems or vice versa.

**Methods:**

The study was part of the Norwegian Mother and Child Cohort Study. The sample consisted of 10 860 toddlers, followed up to age 36 months. We used data from maternal questionnaires from gestation week 17 and at child ages 18 and 36 months, and data from the Medical Birth Registry of Norway. Child height and weight were assessed at child health stations and recorded by mothers. Behavior problems were assessed using shortened subscales from the Child Behavior Checklist. Statistical analyses were conducted using structural equation modeling.

**Results:**

Behavior problems in toddlers were not associated with higher body mass index cross-sectionally at either age 18 or 36 months, and there was no indication that behavior problems caused increasing body mass index over time or vice versa.

**Conclusions:**

The association between behavior problems and body mass index found in older children did not appear in toddlers up to age 36 months. Future studies should focus on the age span from 3 to 6 years, which includes the period of adiposity rebound.

## Background

In recent decades, childhood overweight has been increasing rapidly worldwide. Between 1975 and 2000 in Norway, body weight in childhood increased evenly across the entire weight spectrum of weight by 1 unit of the body mass index (BMI) (kg/m^2^). In 2000, close to 22% of all newborns in Norway weighed more than 4 kg [1]. Today, around 19% of Norwegian children and adolescents age 4-15 years are above the 90^th ^weight-for-height percentile of the growth references for the years 1971-1974 [2]. However, for children younger than age 4, exact overweight rates are still lacking. The rapid increase of overweight rates in children is alarming because of the serious consequences of overweight for health [3,4]. First, obese children are at high risk of obesity in adulthood [3]. Moreover, obesity has adverse effects on many health parameters, such as blood pressure, cholesterol, triglycerides, and insulin resistance. These parameters in turn increase the risk for cardiovascular disorders and type 2 diabetes later in life, among other things [4]. In addition, thickening of the arteries, high blood pressure, fatty streaks in the arteries, and even diabetes type 2, formerly considered exclusively an adult disease, have been observed increasingly during childhood [3,5].

Increasing evidence suggests that child temperament and behavior problems are associated with overweight [6-12]. There are several mechanisms that may account for this association. Children with behavior problems in the externalizing domain may have a relatively underdeveloped capacity for self-regulation [13]. This self-regulatory capacity may in turn impact children's health behaviors (e.g., their diet). Poor self-regulation may prevent children from attending to satiety cues and may prevent them from adequately moderating their eating behaviors. Indirectly, poor self-regulation may prevent children from modulating negative emotionality or emotional reactivity, which may contribute to the development of maladaptive behaviors, such as higher consumption of highly palatable foods - foods containing high amounts of sugar, salt, and fat - and overeating. This "comfort eating" may eventually lead to the emergence of child overweight or obesity [12,14]. In support of this, we found recently that 18-month-old children scoring higher on externalizing problems consumed more sweets and sweet drinks [15]. Especially with infants and toddlers, parents may overfeed difficult children in a misguided effort to calm them or to reduce their emotional intensity [9,16]. Also, a recent study showed that maternal insensitivity, i.e., not being well attuned to the child's wants and needs, was associated with significantly higher risks for the child being overweight or obese [12].

Another mechanism implies that overweight leads to emotional and behavior problems, as overweight individuals may experience a number of frustrations. For instance, obese toddlers may experience that they are physically less able to participate in common activities together with other children or that they are excluded or rejected by them. Indeed, rejection of obese children by their peers has been shown as early as age 3 years [17]. Obese children may also experience that their mothers try to control and restrict their food intake, particularly the intake of highly palatable but unhealthy foods [18]. Mothers have been shown to use controlling feeding practices with children as young as age 1 or 2 years [19,20].

A third reason for the association between behavior problems and overweight may be shared, biologically based constitutional factors. Temperament/behavior traits, taste preferences, and the regulation of appetite are all steered by the dopaminergic system [21-25]. Accumulating evidence indicates that the pathways in the brain governing appetite and emotion regulation are interrelated. Consequently, a common set of factors contribute to both a child's risk for overweight and the degree to which a child displays behavior problems [26].

The mechanisms presented here do not exclude each other: It is possible that there is a reciprocal relationship between behavior problems and weight over time, where behavior problems cause overweight, and overweight in turn causes behavior problems.

To date, however, we know little about the age at which the relationship between behavior and weight first appears. For children below 5 years, there are only four community studies available that examine the cross-sectional association between overweight and behavior [8,10,27,28]. Three of these studies found associations [8,10,28] (although one of them found rather small effect sizes [28]), and one study found none [27]. There are also some prospective studies investigating this relationship. One small laboratory study examined the association between infant temperament, a construct tapping the stable aspects of behavior [29], and early childhood overweight over time. That study found that the temperament dimension labeled "distress to limitations" was positively correlated with percentage of body fat in early childhood [11]. Also Anderson et al. [6] recently demonstrated that externalizing behavior problems were associated with higher BMI and obesity in children as young as age 24 months. This average difference in BMI remained stable through age 12 among the white children in the study. On the other hand, another study following children from 6 months onwards found that children with difficult temperament and insensitive mothers had significantly higher risks for being overweight-or-obese during the school age phase but not during early childhood [12]. Similarly, a different study conducted longitudinal analyses of the relationship between BMI and behavior problems from age 2 years through the 6^th ^grade and found that higher BMI was associated with an increased likelihood for internalizing problems beginning in the 1^st ^grade [7]. One more study showed that boys with a shorter attention span and girls who were difficult to soothe and had more negative mood at age 1 year, had greater increases in standardized weight and were more likely to be overweight or obese at the age of 6 years [9]. No information was given, however, at which age these associations first emerged.

To summarize, the onset of the relationship between behavior and weight remains unclear. Community studies on the association of weight development and temperament or behavior problems in infants and toddlers are sparse. One exception is our previous study of close to 30 000 infants in Norway [30]. That study was the first to examine the association between difficult child temperament (that is, distress, crying, and fussing) and overweight in 6-month-old infants. At that early age, we found no clinically significant relationship between difficultness and overweight.

**Figure 1:**
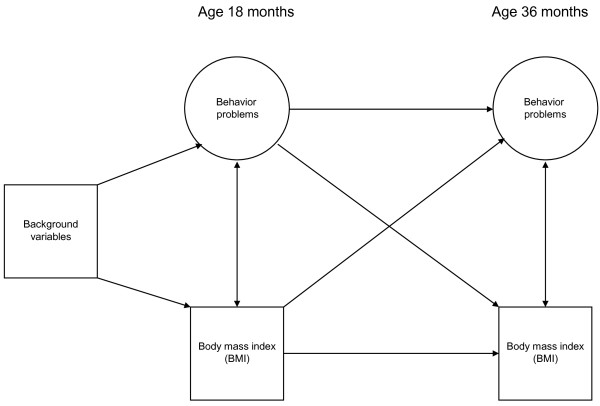
**Conceptual model**. Figure 1 depicts the conceptual model of the study. It shows the cross-sectional and longitudinal relationships (in the time span between age 18 months and 36 months) between behavior problems and BMI which are to be examined. The cross-sectional relations correspond to correlations and are represented by bowed two-sided arrows. The longitudinal relations correspond to regressions and are represented by straight one-sided arrows. Both behavior problems and weight are expected to be associated over time, indicating relative stability. The cross-lagged pathways are depicted by the crossing arrows. All relationships are controlled for relevant confounding variables (background variables).

In the present study we focus on the concurrent and prospective relationship between BMI on the one hand and behavior problems on the other hand in the same cohort between the age of 18 and 36 months. By examining the cross-sectional associations of weight and behavior problems, we can roughly determine the time point at which this association begins to emerge. Moreover, we will examine the cross-lagged pathways between behavior problems and weight over time. This means that we will compare the association between behavior problems at 18 months and weight at 36 months with the association between weight at 18 months and behavior problems at 36 months. In this way, we can examine their co-development over time and thus shed light on their causal sequence. Figure 1 depicts the conceptual model of the study.

## Methods

### Design and Participants

The data collection was conducted as part of the Norwegian Mother and Child Cohort Study (MoBa) at the Norwegian Institute of Public Health. With the target population of all women who give birth in Norway, this national cohort study provides a broad basis to study health development. There are no exclusion criteria, and practically all maternity units in Norway with more than 100 births annually are included. Recruitment started in 1999 and continued up to the end of 2008. The women received a postal invitation to participate in the MoBa study together with their appointment cards for routine ultrasound scans in week 17-18 of gestation. The assessment points are at gestation weeks 17 and 30, and at child ages 6, 18, and 36 months. The participation rate at first assessment was 42.7%. The MoBa study obtained ethical approval by the Regional Committees for Medical and Health Research Ethics, and all women included gave written informed consent to participate. [31].

For the present study, we used questionnaire data from gestation week 17 and from child ages 6, 18, and 36 months and information from the Medical Birth Registry of Norway (MBRN). In Norway, every pregnancy that lasts at least 12 weeks is recorded in the MBRN. The MBRN contains data on parental marital status, the pregnancy, maternal health, the birth, and the child [32].

Information was available on 15 527 toddlers that had reached age 36 months. Because of meaningless values for some variables (such as 22 cm for height), 930 toddlers had to be excluded. In addition, 3 737 toddlers were excluded because of missing data on relevant background variables, such as child sex, maternal BMI, and education. To handle missing data on the child's BMI and the Internalizing and Externalizing Scales, the H1 estimator was used in the estimation procedure using the statistical modeling program Mplus. The H1 estimator estimates missing values on the dependent variables with help of all covariates in the structural model [33]. The final sample for the multivariate analyses consisted of 10 860 toddlers.

### Measures

Information regarding child sex was retrieved from the MBRN. If an entry in the MBRN was missing, maternal report was used instead (at child age 6 months). Information regarding maternal BMI at child age 18 months and maternal education was retrieved from the questionnaires at gestation week 17 and at child age 18 months.

As a part of regular health check-ups, Norwegian children are measured and weighed by well-trained and specialized staff at community-based health check stations. These measurements are recorded on health charts of which the mothers retain a copy. Weight and height at the ages of 18 and 36 months were retrieved from those health charts. Mothers were asked to copy the information from the charts and report it on the questionnaire. We used this information to calculate the BMI for each child at 18 and 36 months of age.

At child ages 18 and 36 months behavior problems were assessed using selected items from the Child Behavior Checklist/11/2-5/LDS (CBCL/11/2-5/LDS) [34]. Item selection was necessary due to questionnaire space restrictions typical for large inter-disciplinary epidemiological studies. The selected items represented subscales of the Internalizing domain ("emotionally reactive," "anxious/depressed," and "somatic complaints") and subscales of the Externalizing domain ("attention problems" and "aggressive behavior") (see Table 1). The item selection procedure based on consensus among specialists in clinical and developmental psychology and aimed at representing each subscale with items that were both clinically and theoretically relevant for Internalizing and Externalizing behavior. Mothers reported the extent to which they agreed with the behavior statements using the following 3-point Likert scale: 1 = not true; 2 = somewhat or sometimes true; 3 = very true or often true. When feasible (not for the singular item representing somatic complaints at child age 18 months), inter-item correlations were used as an estimate of the internal consistency in the subscales. All except one subscale (somatic complaints at child age 36 months) fell in the recommended range of .15 - .50 suggested by Clark and Watson [35]. Moreover, constructing latent factors for the subscales eliminated measurement error and further improved their internal consistency. As a result the majority of the indicators yielded high factor loadings between .50 and .89. Only four of the 29 items yielded moderate loadings ranging between .30 and .50.

**Table 1:** Items from the Child Behavior Checklist/11/2-5/LDS (CBCL/11/2-5/LDS) assessed at child age 18 and 36 months

	18 months	36 months
***Internalizing Subscales***		
I) Emotionally reactive	"Disturbed by any change in routine"	"Disturbed by any change in routine"
		"Rapidly shifts between sadness and excitement"
II) Anxious/depressed	"Clings to adults or too dependent"	"Clings to adults or too dependent"
	"Gets too upset when separated from parents"	"Gets too upset when separated from parents"
	"Too fearful or anxious"	"Too fearful or anxious"
III) Somatic complaints	"Doesn't eat well"	"Doesn't eat well"
		"Constipated; doesn't move bowels"
		"Stomachaches (without medical cause)"
		"Vomiting, throwing up (without medical cause)"

***Externalizing Subscales***		
IV) Attention problems	"Can't concentrate; can't pay attention for long"	"Can't concentrate; can't pay attention for long"
	"Can't sit still; restless or hyperactive"	"Can't sit still; restless or hyperactive"
	"Quickly shifts from one activity to another"	"Quickly shifts from one activity to another"
		"Poorly coordinated or clumsy"
V) Aggressive behavior	"Defiant"	"Defiant"
	"Gets in many fights"	"Gets in many fights"
	"Hits others"	"Hits others"
		"Can't stand waiting; wants everything now"
		"Demands must be met immediately"

### Statistical Analysis

We used structural equation modeling (SEM), a statistical method for estimating the extent to which the data fit a comprehensive model. SEM also supports analyzing prospective data, estimating error variance, and comparing different effects within the same model. The estimations were performed using the statistical software package Mplus 4.2 [33].

We carried out separate analyses for the subscales from the Internalizing and Externalizing domains. Using confirmatory factor analysis [33] we first constructed latent factors for each subscale at both time points (see Table 1 for the items used for each factor). However, due to a very high correlation between the subscales emotionally reactive and anxious/depressed, we represented them in a single latent factor labeled "negative emotionality" in order to obtain an acceptable fit.

In the next step for both models we tested whether the CBCL subscale factors were equivalent at child age 18 and 36 months (test of measurement invariance); this could be confirmed.

We then tested our main model by means of a cross-lagged panel analysis (CLPA) [36]. We examined the cross-sectional associations between the child's BMI and the latent CBCL subscale factors, as well as the longitudinal stabilities and cross-lagged effects of the child's BMI and CBCL subscale factors, controlled for relevant background variables (Figure 1).

## Results

### Descriptives

At the age of 18 months, children's BMI ranged from 13.1 to 20.2 (mean (M) = 16.7, standard deviation (SD) = 1.3). At the age of 36 months, the children's BMI ranged from 12.3 to 19.8 (M = 16.0, SD = 1.4). Based on the BMI cut-off points for overweight and obesity in children between 2 and 18 years provided by Cole and colleagues [37] (85^th ^and 95^th ^percentile of the age- and sex-adjusted BMI distribution across a range of international samples, respectively), only 10.7% of the toddlers of our sample were overweight, and only 0.7% were obese at the age of 36 months.

Mothers' BMI at child age 18 months ranged from 14.7 to 34.5 (M = 24.2, SD = 3.6) and correlated 0.07 and 0.09 with their children's BMI at 18 months and 36 months, respectively. According to the World Health Organization definition [4], 36.2% of the mothers of our sample were overweight (BMI > 25), and 8.6% were obese (BMI > 30). Maternal duration of education ranged from 8 to 18 years (M = 14.5, SD = 2.4).

### Bivariate results

Table 2 shows all unadjusted bivariate correlations between the CBCL subscales and child BMI at ages 18 and 36 months, respectively. The stability of the subscales over time was relatively high. Moreover, all subscales inter-correlated cross-sectionally as well as longitudinally, although the correlation of the subscale somatic complaints with the subscales of the Externalizing domains was somewhat lower. None of the CBCL subscales correlated with child BMI in the expected direction. However, we found that a greater extent of somatic complaints (e.g., "child doesn't eat well") was associated with lower, not higher, child BMI both cross-sectionally and longitudinally.

**Table 2:** Unadjusted bivariate correlations between CBCL subscales and BMI at child age 18 and 36 months

	**1**.	**2**.	**3**.	**4**.	**5**.	**6**.	**7**.	**8**.	**9**.	**10**.
***18 Months***										
1. Child BMI	1.00									
2. Negative emotionality	-0.07	1.00								
3. Somatic complaints	-0.36	0.43	1.00							
4. Attention problems	-0.02	0.40	0.22	1.00						
5. Aggressive behavior	0.04	0.44	0.17	0.43	1.00					

***36 Months***										
6. Child BMI	0.58	-0.04	-0.27	0.00	0.03	1.00				
7. Negative emotionality	-0.04	0.59	0.25	0.36	0.31	-0.05	1.00			
8. Somatic complaints	-0.22	0.31	0.43	0.23	0.15	-0.22	0.55	1.00		
9. Attention problems	-0.01	0.29	0.17	0.62	0.34	0.01	0.57	0.34	1.00	
10. Aggressive behavior	0.02	0.30	0.14	0.39	0.49	0.02	0.61	0.32	0.64	1.00

### Multivariate results

**Figure 2:**
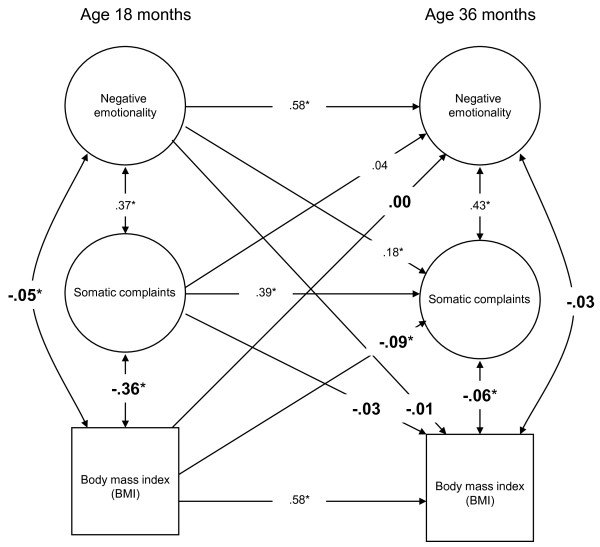
**Structural model for the Internalizing domain**. Figure 2 shows the results of the multivariate analyses for the CBCL subscale factors from the Internalizing domain in the time span between age 18 months and 36 months. All cross-sectional and longitudinal relationships are reported. Double-headed arrows represent correlations, one-sided arrows represent regressions. Coefficients include correlation coefficients and standardized betas. Controlling the initial association between Internalizing behavior problems and BMI, the results show the extent to which Internalizing behavior problems are related with subsequent BMI, and whether BMI is associated with subsequent internalizing behavior problems (cross-lagged pathways). The associations pertaining to the relationships between the different domains of Internalizing behavior problems and BMI are depicted in larger and bold numbers. All associations are adjusted for the background variables child sex, maternal BMI at child age 18 months, and maternal education.

**Figure 3:**
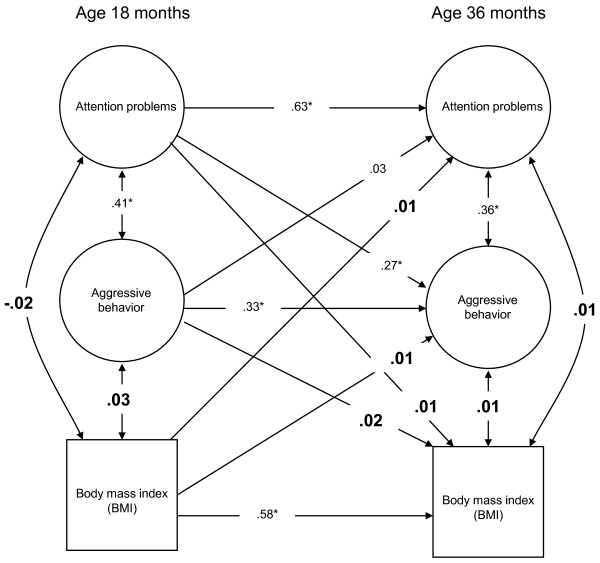
**Structural model for the Externalizing domain**. Figure 3 shows the results of the multivariate analyses for the CBCL subscale factors from the Externalizing domain in the time span between age 18 months and 36 months. All cross-sectional and longitudinal relationships are reported. Double-headed arrows represent correlations, one-sided arrows represent regressions. Coefficients include correlation coefficients and standardized betas. Controlling the initial association between Externalizing behavior problems and BMI, the results show the extent to which Externalizing behavior problems are related with subsequent BMI, and whether BMI is associated with subsequent Externalizing behavior problems (cross-lagged pathways). The associations pertaining to the relationships between the different domains of Externalizing behavior problems and BMI are depicted in larger and bold numbers. All associations are adjusted for the background variables child sex, maternal BMI at child age 18 months, and maternal education.

Figures 2 and 3 show the results of the multivariate analyses, separated according to the Internalizing and Externalizing domains and adjusted for child sex and maternal BMI and education. Both models showed a good fit, as measured by Root Mean Square Error of Approximation (RMSEA) = 0.02, Tucker Lewis Index (TLI) = 0.95, and Comparative Fit Index (CFI) = 0.95 for the Internalizing factors; and RMSEA = 0.05, TLI = 0.94 and CFI = 0.93 for the Externalizing factors. RMSEA estimates the degree of misspecification of the model in the population, and the indices TLI and CFI are different measures of how much better the model fits the data as compared with a baseline model where no relationships are assumed. According to Hu et al., a model fits well if the RMSEA is 0.06 or lower and TLI and CFI are equal to or greater than 0.95 [38].

The latent factor somatic complaints from the Internalizing domain was markedly negatively correlated with the BMI at child age 18 months as suggested by the unadjusted bivariate results but much less so at child age 36 months. This indicates that there is no co-development between BMI and somatic complaints over and beyond their initial association at 18 months. Further, there was no association between the other latent factor from the Internalizing domain, negative emotionality, and BMI at any time point. Similarly, the latent factors attention problems and aggressive behavior from the Externalizing domain had no noteworthy cross-sectional associations with the child's BMI.

In the next step, we probed whether the prospective cross-lagged effects from behavior problems at 18 months to child BMI at 36 months were equal to those from child BMI at 18 months to behavior problems at 36 months. This was carried out by testing a model where the cross-lagged effects were forced to be equal against a model where the cross-lagged effects were freely estimated. The models were not significantly different (negative emotionality: Δ*χ^2 ^= *0.36, Δ*df = 1, p = *0.55; somatic complaints: Δ*χ^2 ^= *0.00, Δ*df = 1, p = *0.96; attention problems: Δ*χ^2 ^= *0.01, Δ*df = 1, p = *0.91; aggressive behavior: Δ*χ^2 ^= *0.52, Δ*df = 1, p = *0.47), suggesting that there is no difference in the size of the cross-lagged effects. Both cross-lagged effects were negligible, showing that child behavior and child BMI did not influence each other over time.

## Discussion

The aim of this study was to investigate the concurrent and prospective relationship between BMI and behavior problems in toddlers aged 18 to 36 months. Based on biological reasoning as well as findings from earlier studies [8,10,11,14-25,28,29,39-51], we expected Internalizing and Externalizing behavior problems to occur more often in children with higher weight. Moreover, we expected to find a co-development of weight and behavior problems, where both weight and behavior problems increase each other over time in a prospective vicious circle.

However, we found neither cross-sectional nor prospective associations. The only association that we found between somatic complaints and BMI was the opposite of the association that we expected. Indeed, children with more somatic complaints had a lower BMI than children with fewer complaints. However, this is not very surprising, as the somatic complaint items that are an integral part of Internalizing problems mostly tapped eating behavior and digestion problems. Constipation and stomachaches are likely to reduce food intake and thus to lead in turn to comparatively lower weight.

There may be several explanations for the lack of associations between Internalizing and Externalizing behavior problems (except somatic complaints) and weight. An important reason may be the young age of our sample. Even if children with externalizing or internalizing problems are inclined to eat more obesogenic foods [15], it may take a longer time before a diet high in obesogenic foods leads to overweight or obesity. In a similar vein, negative reactions by mother or peers may occur less frequently at age 18 months, an age when many mothers still prefer a chubby child to a thin child [52]. Indeed, there has only been a single, small study that found negative attitudes and behaviors towards overweight 3-year-olds [17]. Furthermore, whereas the association between overweight/obesity and psychological problems is quite well established in older children and adults [11,39,47,53-55], the findings for children below 5 years are ambiguous [8,10,27,28]. In addition, one could argue that small children's eating behavior is not under their own control but largely determined by their family and cultural environment [56]. However, although family environment determines the choice of foods a child is exposed to, infants and toddlers eat what they like and spit out what they dislike.

Readers should note some limitations to our findings. Measuring behavior problems using shortened versions of the established questionnaires might threaten the construct validity of the Internalizing and Externalizing scales. Nonetheless, internal consistency was satisfactory for the majority of the subscales [35]. By using latent factors based on confirmatory factor analyses, we eliminated measurement error and improved the internal consistency of the constructs even more. Moreover, demonstrating their discriminant validity we showed in previous publications that the Internalizing and Externalizing scales, measured conventionally, correlated with other variables such as maternal smoking and child diet in a meaningful way [15,57]. Yet, we cannot exclude that we may have measured somewhat narrower constructs than those assessed by the original CBCL. Particularly the subscale "somatic complaints" can raise questions considering its validity in the given context. The selected items such as "Doesn't eat well" (see Table 1) are closely linked to the child's actual diet, metabolism, and weight. Therefore, a negative association with BMI seems unsurprising. In fact, other studies also cautioned that real physical symptoms may be confused with the symptomatology that reflects psychosocial disturbance when applying the subscale "somatic complaints" [58]. Still, somatic complaints belong to the core symptoms of internalizing problems [59]. Particularly in small children, who have limited cognitive and language abilities, somatic complaints can serve as a crucial hint for internalizing problems. We therefore chose to keep this subscale in our analyses.

Another limitation concerns the measurement of child weight and height. Generally, the most accurate way to determine overweight or obesity is to measure skinfold thickness at different locations (triceps, subscapularis) [2]. However, this type of measurement in parallel with anthropometric measures is not feasible for a population study including 100 000 children. The weight and height measures on which maternal reports based were carried out in a standard fashion by specialized health care staff. However, the large number of missing values suggests that many mothers did not have their child's health chart at hand when filling in the questionnaires. Other mothers may have made errors when copying from the health charts. Yet, we believe that these errors were few and random. When we compared birth weight and height recorded in the Medical Birth Registry with maternal reports of birth weight and height on the 6 months questionnaire, we found correlations of .99 and .96 respectively, indicating that mothers' report of their children's' weight and height in MoBa is accurate.

Another limitation of our study pertains to external validity. With the relative low recruitment of 42.7% in the Norwegian Mother and Child Cohort Study (MoBa), selection bias is likely. Possibly, women of higher socioeconomic status are overrepresented in the study [31]. Thus, the study might not be representative for the entire spectrum of the Norwegian population. However, new results from MoBa suggest that even though selected estimates of exposures and outcomes were biased, exposure-outcome associations were not [60]. Therefore, it is reasonable to assume that our results hold in the entire population, too.

## Conclusions

Despite these limitations, this study fills an important gap in the literature by suggesting that behavior problems and weight are still unrelated in toddlers up to age 3. This finding is substantial, as it bases on a large nationwide sample, examines behavior on a fine-grained level, and uses advanced statistical analyses that are suitable to detect even minor associations. Because the association of behavior problems with overweight has been found in 5- to 6-year-olds, further studies should focus on the age span between 3 and 6 years, which also includes the period of adiposity rebound (when the BMI normally increases after having declined to a minimum), which is "a critical period for the development of obesity" [61].

## Competing interests

The authors declare that they have no competing interests.

## Authors' contributions

SGN designed the study, performed the statistical analyses, and drafted the manuscript. KAH participated in the design of the study, helped with the statistical analyses, and was involved in revising the manuscript content. MEV conceived of the study, participated in its design, and helped to draft the manuscript. All authors approved the final manuscript.

## Pre-publication history

The pre-publication history for this paper can be accessed here:

http://www.biomedcentral.com/1471-2458/10/626/prepub
